# Functional Characterization and Evolutionary Analysis of Glycine-Betaine Biosynthesis Pathway in Red Seaweed *Pyropia yezoensis*

**DOI:** 10.3390/md17010070

**Published:** 2019-01-21

**Authors:** Yunxiang Mao, Nianci Chen, Min Cao, Rui Chen, Xiaowei Guan, Dongmei Wang

**Affiliations:** 1 Key Laboratory of Marine Genetics and Breeding (Ocean University of China) Ministry of Education, Qingdao 266003, China; yxmao@ouc.edu.cn (Y.M.); 13969671852@163.com (N.C.); caominjiayou@163.com (M.C.); cherryruirui99@163.com (R.C.); guanxw1995@163.com (X.G.); 2 Laboratory for Marine Biology and Biotechnology, Qingdao National Laboratory for Marine Science and Technology, Qingdao 266237, China; 3 College of Marine Life Sciences, Ocean University of China, Qingdao 266003, China

**Keywords:** *Pyropia yezoensis*, desiccation, betaine, PEAMT, CDH, BADH

## Abstract

The red seaweed *Pyropia yezoensis* is an ideal research model for dissecting the molecular mechanisms underlying its robust acclimation to abiotic stresses in intertidal zones. Glycine betaine (GB) was an important osmolyte in maintaining osmotic balance and stabilizing the quaternary structure of complex proteins under abiotic stresses (drought, salinity, etc.) in plants, animals, and bacteria. However, the existence and possible functions of GB in *Pyropia* remain elusive. In this study, we observed the rapid accumulation of GB in desiccated *Pyropia* blades, identifying its essential roles in protecting *Pyropia* cells against severe osmotic stress. Based on the available genomic and transcriptomic information of *Pyropia*, we computationally identified genes encoding the three key enzymes in the GB biosynthesis pathway: phosphoethanolamine *N*-methyltransferase (PEAMT), choline dehydrogenase (CDH), and betaine aldehyde dehydrogenase (BADH). *Pyropia* had an extraordinarily expanded gene copy number of CDH (up to seven) compared to other red algae. Phylogeny analysis revealed that in addition to the one conservative CDH in red algae, the other six might have originated from early gene duplication events. In dehydration stress, multiple CDH paralogs and PEAMT genes were coordinating up-regulated and shunted metabolic flux into GB biosynthesis. An elaborate molecular mechanism might be involved in the transcriptional regulation of these genes.

## 1. Introduction

Plant performance and yield responses to water deficit stress conditions have been extensively studied [1,2,3]. Water deficiency causes cell metabolic disorder and cell membrane mechanical damage. A decline in leaf relative water content (RWC) reflects a loss of turgor that results in limited cell expansion and thereby reduced growth in crop plants [4,5]. Glycine betaine (GB) (*N*,*N*,*N*-trimethylglycine) is a quaternary ammonium compound found in bacteria, halophilic archaebacteria, marine invertebrates, plants, and mammals [6,7,8,9]. As an organic osmolyte, GB can maintain the osmotic balance and stabilize the quaternary structure of complex proteins under abiotic stresses. The protective role of GB has been widely reported in plants [10,11,12]. Treating plants with exogenous GB or increasing GB content in transgenic plants would greatly enhance their tolerance to osmotic stresses. Under chilling-stressed conditions, the heterologous expression of the betA gene significantly increased the tolerance of transgenic cotton seedlings to chilling injury through the accumulation of high levels of GB [13]. GB is also known to stabilize both photosystem II complexes and Rubisco under abiotic stress.

Four biosynthetic pathways for GB are known, three involve the oxidation of choline and one is the methylation of glycine [14,15]. In the choline oxidation pathway, GB is synthesized through a two-step oxidation of choline [16]. The first step converts choline into betaine aldehyde and is catalyzed by Rieske-type iron-sulphur enzyme choline monooxygenase (CMO; EC:1.14.15.7) in plants [17] and membrane-associated choline dehydrogenase (CDH; EC:1.1.99.1) in *E.coli* [18], animals, and *Thalassiosira pseudonana* [19]. The second step is catalyzed by nicotinamide adenine dinucleotide (NAD)-dependent betaine aldehyde dehydrogenase (BADH; EC:1.2.1.8) for the final biosynthesis of betaine. In soil bacterium *Arthrobacter globiformis*, betaine is directly catalyzed by choline oxidase (COD; EC:1.1.3.17) to degenerate hydrogen from choline. Besides this, three sequential methylation steps of phosphoethanolamine to generate phosphocholine, catalyzed by phosphoethanolamine *N*-methyltransferase (PEAMT; EC:2.1.1.103), are also reported to be the rate limiting step in the choline-biosynthetic pathway [20,21]. The alternative GB biosynthetic pathway involves the three-step methylation of glycine, which is reported in halophilic microorganisms and also the diatom *T. pseudonana* [22,23]. 

*Pyropia yezoensis*, previously known as *Porphyra yezoensis* [24], Rhodophyta, is a typical intertidal macroalgae. During daily low tides, *P. yezoensis* is routinely exposed to high levels of light, dehydration, and extreme fluctuations in temperature and osmotic pressure due to the seawater-to-air transition [25]. The blades can tolerate dehydration with a water loss of up to 85%, and recover physiological activities immediately upon rehydration [26]. These characteristics make *P. yezoensis* an ideal model for studying the molecular mechanisms of stress tolerance in intertidal-zone seaweeds. However, to date, there have been few reports investigating the cellular osmolytes in *P. yezoensis*. Is GB present in *Pyropia* cells? What are the roles of the GB in response to osmotic stress in *Pyropia*? What influences the diversity and conservation of the GB biosynthesis pathway in *Pyropia*? To address these questions, in this study, we computationally identified genes involved in the betaine biosynthesis pathway. We identified the variation of enzymatic activities and transcriptions for key genes during dehydration as well as re-hydration processes.

## 2. Results

### 2.1. GB Contents in Dehydrated P. yezoensis Blades 

**Figure 1:**
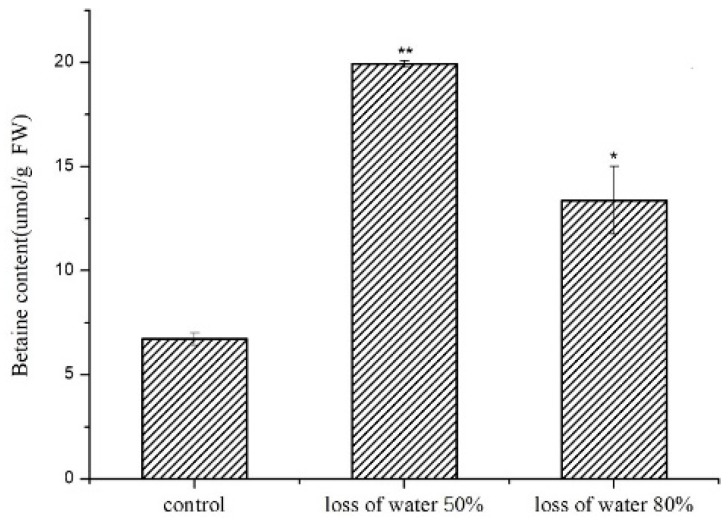
Profiles of glycine betaine (GB) contents in *P. yezoensis* blades under de-hydration stress. The seaweeds were desiccated until the total water content reached 50% and 80%. Three replicates were produced for each treatment. Error bars indicate standard errors. The statistical significance compared to the control sample is indicated with one or two asterisks (*p* value is lower than 0.05 (*) or 0.01 (**), respectively).

To determine whether GB exists in red seaweed *P. yezoensis* and whether it is involved in desiccation tolerance, we detected the amounts of GB in the dehydration process. In 40-day old *P. yezoensis* blades, 6.720 μmol/g (fresh weight, FW) of GB was detected. The amount increased sharply to 19.924 μmol/g FW (a 2.96-fold increase compared to the control) when 50% of water was lost in the blades (Figure 1). However, after an extension of dehydration with water loss at 80%, the GB amount dropped slightly, although there was still a 1.990-fold increase compared to the basal level. 

### 2.2. GB Biosynthesis Pathway in P. yezoensis 

The biosynthesis pathway of GB exhibited great diversity in plants, animals, bacteria as well as unicellular diatoms. Mainly two metabolic pathways lead to the GB biosynthesis, namely the choline oxidation pathway and the glycine methylation pathway. To identify candidate genes encoding the key enzymes related to GB biosynthesis in *P. yezoensis* and to further refer to its evolutionary history, we searched genomic and transcriptomic information published for *P. yezoensis* and other algal genomes including primary endosymbionts green algae and red algae, as well as the secondary endosymbionts diatom and *Ectocarpus*, via a blast program using the corresponding homologous genes from the above organisms as queries.

**Figure 2:**
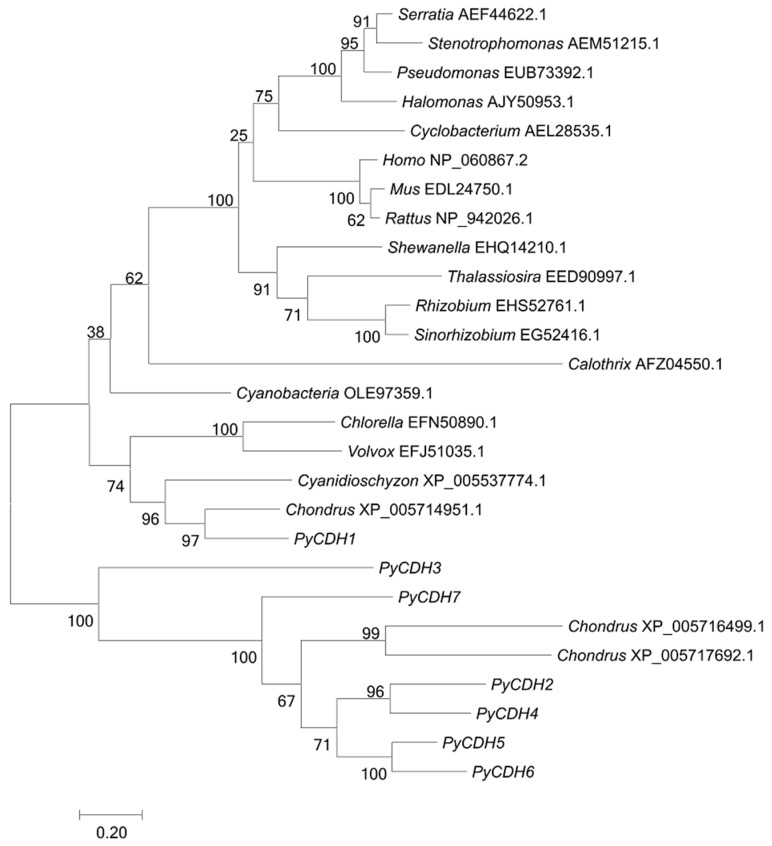
Phylogenetic analysis of *PyCDH*s and other choline dehydrogenase (CDHs). An unrooted phylogenetic tree was constructed using the maximum likelihood phylogenetic method in the MEGA version 7.0 software. The branch lengths are proportional to the evolutionary distances. The sequence of CDH genes include *Cyanobacteria bacterium* 13_1_20CM_4_61_6 (OLE97359.1), *Thalassiosira pseudonana* CCMP1335 (EED90997.1), *Volvox carteri f. nagariensis* (EFJ51035.1), *Calothrix* sp. PCC 6303 (AFZ04550.1), *Cyanidioschyzon merolae* strain 10D (XP_005537774.1), *Chlorella variabilis* (EFN50890.1), *Cyclobacterium marinum* DSM 745 (AEL28535.1), *Sinorhizobium meliloti* AK83 (AEG52416.1), *Stenotrophomonas maltophilia* JV3 (AEM51215.1), *Serratia plymuthica* AS9 (AEF44622.1), *Rhizobium* sp. PDO1-076 (EHS52761.1), *Halomonas* sp. KO116 (AJY50953.1), *Shewanella baltica* OS183 (EHQ14210.1), *Pseudomonas* sp. GM41(2012) (EUB73392.1), *Homo sapiens* (NP_060867.2), *Mus musculus* (EDL24750.1), *Rattus norvegicus* (NP_942026.1), and *Chondrus crispus* (XP_005714951.1, XP_005717692.1, XP_005714951.1).

In the choline oxidation pathway, the first enzyme that catalyzes the oxidation of choline into choline aldehyde is choline dehydrogenase (CDH). In *P. yezoensis*, we found seven genes encoding proteins with similarity to CDH homologs, while only three and one were found in *Chondrus* and the unicellular red algae *Cyanodioschyzon*, respectively. All of them harbored a GMC-oxred N conserved domain at the *N*-terminus (Table 1). We carried out the phylogenetic analysis to probe the evolutionary forces driving the expansion of CDH genes. In the evolutionary tree, the algal CDHs are grouped together, while the bacteria and animal homologs are in two other clusters, suggesting an ancient separation in the evolution of algal CDHs. The *PyCDH1* showed a phylogeny with strong affiliation with its red-algal homologs. The other six *PyCDHs* formed a sister group with two homologs from *Chondrus* (Figure 2). The most plausible explanation for the extraordinary expansion of CDH genes was the explosive gene duplication events in the *P. yezoensis* genome. 

**Figure 3:**
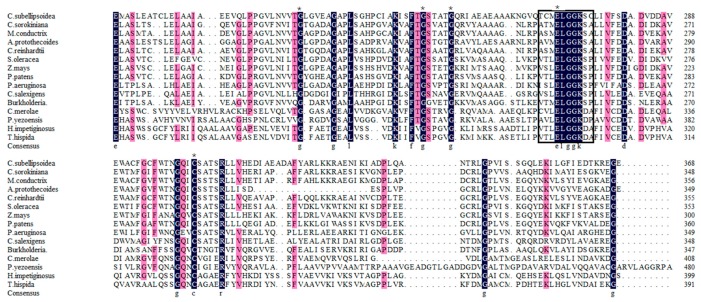
Multiple sequence alignment of betaine aldehyde dehydrogenase (BADH) amino acid sequences by using Clustal X 2.0. The same amino acid residue is indicated in black font. Asterisks indicate putative residues involved in nicotinamide adenine dinucleotide (NAD^+^) binding, and boxes indicate highly conserved regions in the catalytic sites of various dehydrogenases. The sequence of BADH genes includes: *Coccomyxa subellipsoidea* C-169(XP005645499.1), *Chlorella sorokiniana* (PRW59377.1), *Micractinium conductrix* (PSC76875.1), *Auxenochlorella protothecoides* (XP011398091.1), *Chlamydomonas reinhardtii* (XP001699134.1), *Spinacia oleracea* (AAB41696.1), *Zea mays* (NP001157804.1), *Physcomitrella patens* (XP001756623.1), *Chromohalobacter salexigens* (YP574889.1), *Burkholderia* sp. MR(KIG10012.1), *Cyanidioschyzon merolae* strain 10D(XP005536893.1), *Porphyra yezoensis*, *Handroanthus impetiginosus* (PIN07780.1), *Pseudomonas aeruginosa* (ACD38844.1), and *Tamarix hispida* -2(AIL24124.1).

**Figure 4:**
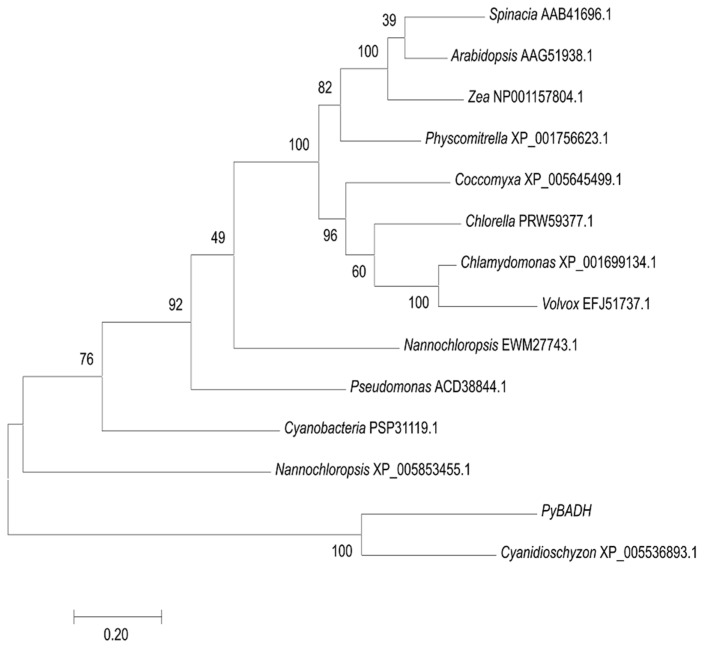
Phylogenetic analysis of *PyBADH* and other betaine aldehyde dehydrogenase (BADHs). An unrooted phylogenetic tree was constructed using the maximum likelihood phylogenetic method in the MEGA version 7.0 software. The branch lengths are proportional to the evolutionary distances. The sequence of BADH genes includes *Spinacia oleracea* (AAB41696.1), *Pseudomonas aeruginosa* (ACD38844.1), *Zea mays* (NP001157804.1), *Physcomitrella patens* (XP001756623.1), *Chlamydomonas reinhardtii* (XP001699134.1), *Coccomyxa subellipsoidea* C-169 (XP005645499.1), *Chlorella sorokiniana* (PRW59377.1), *Cyanidioschyzon merolae* strain 10D (XP005536893.1), *Cyanobacteria bacterium* SW_4_48_29 (PSP31119.1), *Nannochloropsis gaditana* (XP_005853455.1,EWM27743.1), *Thalassiosira pseudonana* (ACI64514.1), *Volvox carteri f. nagariensis* (EFJ51737.1), and *Arabidopsis thaliana* (AAG51938.1).

BADH catalyzes the last step converting betaine aldehyde into betaine. Thus, we also computationally identified the putative BADH genes in *P. yezoensis*. *PyBADH* contains an ORF of 2428 bp, encoding 691 amino acids. *PyBADH* protein harbors a conservative motif x-x-x-E-L-G-G-K-x-x (Figure 3), which is characterized as dehydrogenation, and several residues that are characterized as being involved in NAD^+^ binding and catalytic sites [27]. The phylogenetic analysis of BADHs showed that the *PyBADH* formed a close clade with unicellular red algae homologs (Figure 4). 

PEAMT catalyzes the methylation of phosphoethanolamine to form choline, and is characterized as being essential for betaine biosynthesis in plants. In *P. yezoensis*, we found one putative PEAMT gene encoding 468 amino acids. The functional domain analysis of *PyPEAMT* showed that PEAMT belongs to the *N*-methyltransferase superfamily. 

**Figure 5:**
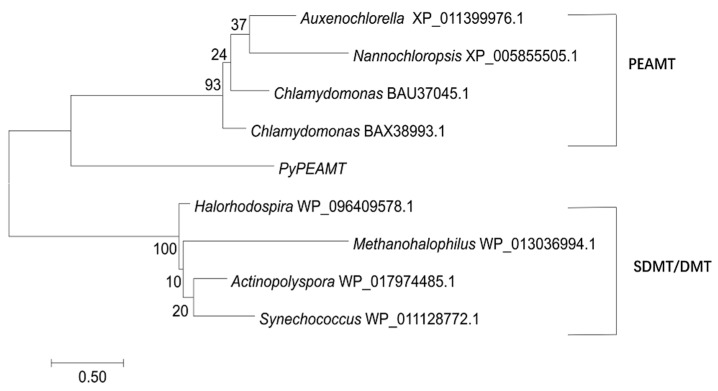
Phylogenetic analysis of *PyPEAMT*, other phosphoethanolamine N-methyltransferase (PEAMTs) and other glycine sarcosine methyltransferase (GSMT). An unrooted phylogenetic tree was constructed using the maximum likelihood phylogenetic method in the MEGA version 7.0 software. The branch lengths are proportional to the evolutionary distances. The sequence of relative genes includes *Auxenochlorella protothecoides* (XP_011399976.1), *Nannochloropsis gaditana* CCMP526 (XP_005855505.1), *Chlamydomonas applanata* (BAX38993.1), *Chlamydomonas asymmetrica* (BAU37045.1), *Actinopolyspora halophila* (WP_017974485.1), *Halorhodospira halochloris* (WP_096409578.1), *Methanohalophilus mahii* (WP_013036994.1), and *Synechococcus* sp. (WP_01128772.1).

In the glycine methylation pathway for GB biosynthesis, the sequential methylation is catalyzed by two AdoMet dependent methyltransferases in bacteria: glycine sarcosine methyltransferase (GSMT) and sarcosine dimethyl-glycine methyltransferase (SDMT). When blasting against *P. yezoensis* unigenes using bacteria homologs as the query, the best hit goes to *PyPEAMT*, as all of them have the conserved methyltransferase domain. However, in the phylogeny tree with both SDMT/DMT and PEAMT homologs included, the *PyPEAMT* was grouped in the PEAMT clade (Figure 5). Therefore, we deduced that the glycine methylation pathway might not exist in the *P. yezoensis* genome. 

### 2.3. Transcriptional Variations of CDH, BADH, and PEAMT Genes Under Dehydration

**Figure 6:**
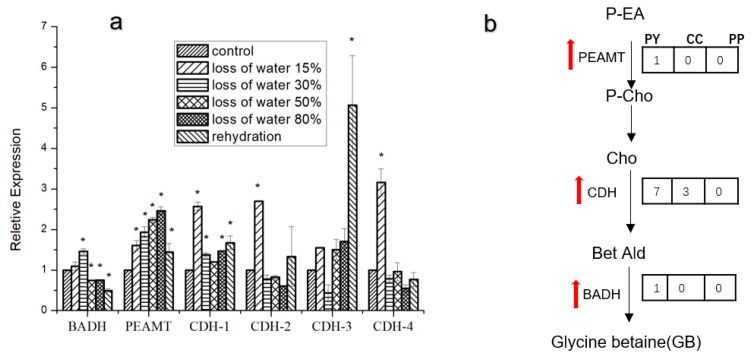
Profiles of putative GB biosynthetic genes. (**a**) Three replicates were produced for each treatment. The symbol * represents significant differences from control values at *p* < 0.05 (Student’s *t*-test). (**b**) P-EA, phosphor-ethanolamine; P-Cho, phosphocholine; Cho, choline; Bet Ald, betaine-aldehyde; PY, *Pyropia yezoensis*; CC, *Chondrus crispus*; PP, *Porphyridium purpureum*. The red arrows indicate the transcriptional up-regulation under desiccation stress. The numbers in the squares refer to the gene copy numbers of each enzyme in *Pyropia yezoensis*, *Chondrus crispus*, and *Porphyridium purpureum,* respectively.

We carried out real-time fluorescence quantitative polymerase chain reaction (PCR) to investigate the transcriptional variation of PEAMT, BADH, and four CDH genes in *P. yezoensis* during the dehydration process. PEAMT exhibited a gradual up-regulation with the aggravation of water loss, and reached a peak at 80% water loss. A synchronized sharp increase in transcription at the onset of dehydration was observed in all the four CDH genes. Although they returned to the basal level thereafter, CDH1, CDH2, and CDH3 increased in the subsequent re-hydration, with CDH3 even rising to a 5.06-fold increase compared to the control. BADH only showed slight up-regulation at 30% water loss. Its transcription dropped thereafter and even went to half of the basal level in re-hydration (Figure 6a). 

### 2.4. CDH Activity under Dehydration Stress in P. yezoensis Seaweeds

**Figure 7:**
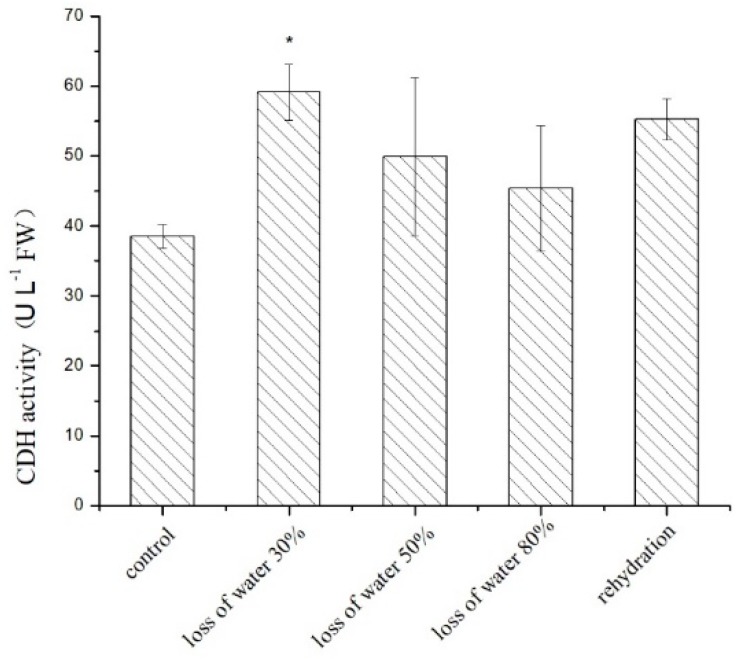
Profiles of CDH activity under dehydration stress in *P. yezoensis*. The algae reached water loss levels of 30 ± 5%, 50 ± 5%, and 80 ± 5%, respectively, as well as rehydration after 80% water loss for 30 min. Three replicates were produced for each treatment. The symbol * represents significant differences from control values at *p* < 0.05 (Student’s *t*-test).

We further detected the enzymatic activity of *P. yezoensis* CDHs in desiccated blades and found that its variation was well correlated with the transcriptional levels. The CDH enzymatic activity increased immediately at the early stage of water loss (1.54-fold) and then slightly dropped to the basal level under severe dehydration. When rehydrated, the enzyme activity increased 1.4-fold again (Figure 7).

## 3. Discussion

The data presented in this study strongly indicates that glycine betaine, as a compatible solute, plays essential roles in protecting *P. yezoensis* against osmotic stress. This is the second algal species (the first red algae) reported to have a betaine biosynthesis pathway. However, in the diatom *T. pseudonana,* the enzymatic activity of CDH was not provided [19]. We also computationally identified homologous genes of PEAMT and BADH in other three red algae as well as green algae (Figure 6b). The wide occurrence of the choline oxidation pathway in organisms strongly suggests its evolutionary conservation. Conversely, the GB methylation pathway is present only in bacteria and the diatom *T. pseudonana,* as reported recently. The PEAMT gene in *P. yezoensis* has high protein similarity to bacteria SDMT and was doubted to be the putative SDMT homolog catalyzing the methylation of glycine. However, the phylogeny analysis did not support this argument. Moreover, at least two proteins (GSMT, SDMT/DMT) or one integrative peptide with two substrate-specific methyltransferase domains such as in *T. pseudonana* are required by the three-step methylation of glycine [19], while only one domain was found in *PyPEAMT*. Therefore, we deduced that the glycine methylation pathway is bacteria-specific and the diatom *T. pseudonana* might obtain this pathway occasionally via horizontal gene transfer, transposable elements, etc. 

Choline, the precursor of betaine, could also go into either the lipid metabolic pathway or the acetylcholine biosynthesis. Choline dehydrogenase was the key enzyme to determine the metabolic direction of choline into betaine biosynthesis. In the *P. yezoensis* genome, up to seven CDH paralogs were identified, representing the highest gene dose among known animal, algal, and bacterial genomes. The increase in particular gene families was frequently reported in genomic analysis. Usually, several mechanisms have been proposed to play essential roles in obtaining new genetic materials for environmental adaptation, including genome-scale duplication, regional duplication due to homologous recombination, and illegitimate recombination, as well as horizontal gene transfer from bacteria or archaea. No evidence of genome duplication was observed in *P. yezoensis*. Besides, in the phylogeny analysis of *PyCDH*s, they were clustered separately from bacteria CDH homologs, excluding the existence of horizontal gene transfer (HGT) events in the CDH expansion. Therefore, according to the close phylogenetic relationship of the six *PyCDH* paralogs, especially the high sequence similarities in the two paralogous groups (PyCDH2 and PyCDH4, PyCDH5 and PyCDH6), we deduced that the most plausible explanation was that multiple gene duplication events leading to the acquirement of new CDH genes happened in the evolution of the *P. yezoensis* genome, accounting for the rapid accumulation of betaine for cell protection when encountering osmotic stresses. 

Another prominent observation in *P. yezoensis* GB biosynthesis was that CDH paralogs exhibited simultaneous up-regulation at the onset of osmotic stress, and then dropped to the basal level when dehydration was ongoing. Three of them also showed transcriptional elevation in re-hydration. The coordinated transcriptional pattern suggested the existence of an elaborate regulatory mechanism that could be induced by osmotic stress. In addition to this, our analyses showed an increase in the enzyme activity for CDH under moderate and severe osmotic stress in *P. yezoensis*. Further study of the cis-regulatory elements or conserved DNA motifs located in their promoter regions would help to illustrate the key components and their regulatory network in response to osmotic stresses.

## 4. Materials and Methods 

### 4.1. Algal Material and Stress Treatment

The pure line RZ58 of *P. yezoensis*, established by clonal cultivation of an isolated single somatic cell and self-fertilization in the laboratory, was used for the experiments. Fresh leafy gametophytes were cultured in bubbling natural seawater with Provasoli’s enrichment solution medium (PES) under 50 μmol photons m–2s–1 at 10 ± 1 °C and a 12:12 light (dark photoperiod before use). The seawater was bubbled continuously with filter-sterilized air and renewed every three days.

*P. yezoensis* blades were subjected to dehydration by exposing them to the air for a period of time. They were collected when water loss was weighed to be 30 ± 5%, 50 ± 5%, and 80 ± 5%, respectively. For rehydration, severely dehydrated samples (water loss at 80 ± 5%) were transferred back into seawater, and collected 0.5 h later. Water loss was determined according to the method by Kim et al. [28]. Three biological replicates were produced for treatment. After harvesting and weighing, the samples were immediately frozen in liquid nitrogen and stored individually at −80 °C until use.

### 4.2. RNA Isolation and cDNA Synthesis

Total RNA was extracted from the thallus using the Plant RNA Kit (Omega, U.S.) in accordance with the manufacturer’s instructions. RNA degradation and contamination were monitored on 1% agarose gels. Purity was evaluated using a NanoPhotometer spectrophotometer (IMPLEN, CA, U.S.). RNA concentration was measured using a Qubit RNA Assay Kit and Qubit 2.0 Fluorometer (Life Technologies, CA, U.S.). RNA integrity was assessed using an RNA Nano 6000 Assay Kit and the Bioanalyzer 2100 system (Agilent Technologies, CA, U.S.). The cDNA templates were synthesized using a First Strand cDNA Synthesis Kit (Roche, Germany) according to the manufacturer’s instructions. Subsequently, 1 μg total RNA was used to synthesize the first-strand cDNA. The resulting cDNA mixture was diluted 10 times by adding nuclease-free water and stored at −20 °C.

### 4.3. Quantitative Real-Time PCR (RT-PCR)

Sequence-specific primers of *PyBADH*, *PyPEAMT*, and *PyCDH*s, based on their multiple alignments, were designed using the Primer 5.0 software (Premier Biosoft International, USA) (Table 2). Quantitative real-time PCR (RT-qPCR) was performed with Light Cycler480 (Roche, Germany). *PyUBC* was simultaneously used as internal control to normalize the amount of messenger RNA (mRNA) in each reaction. The cycling parameters were as follows: 95 °C for 5 min, followed by 45 cycles of 95 °C for 10 s, 60 °C for 10 s, and 72 °C for 20 s. The mean amplification efficiency of each primer pair was checked with the LightCycle^®^480 gene scanning software version 1.5 (Roche, Germany). The 2−ΔΔCt method was used to assess the expression of relative genes [29]. The qPCR results shown are the average (±SD) of three biological repeats.

### 4.4. GB Extraction and Liquid Chromatography Analysis

About 200 mg of the material was ground with liquid nitrogen, then pre-cooled with 1 mL methanol, and ultrasonically extracted for 30 min. This was centrifuged for 10 min at 10,000× *g* (HITACHI, Tokyo, Japan) and the supernatant was mixed after repeating twice, concentrated to dry under a nitrogen blower (NDK200-2, Miulab, Hangzhou, China), dissolved by vortex oscillation in 1 mL water was added, and then filtered with 0.22-µm-pore filter membranes (Millipore).

### 4.5. Detecting Content of GB

The content of betaine was determined by HPLC (L-3000, RIGOL, Beijing, China). By comparing the peak area of the samples with that of the standards (Shanghai yuanye, Bio-Technology, Shanghai, China), the content of the samples was calculated. 

### 4.6. Determination of Choline Dehydrogenase Activity

The choline dehydrogenase activity was detected using a Plant CHDH ELISA kit (Mlbio, Shanghai, China). Phosphate buffer saline (PBS) (100 μg material, 900 μL PBS) was added into a tube after grinding samples with liquid nitrogen. The samples were mixed and then centrifuged for 20 min (Hitachi, Tokyo, Japan) at 2500× *g* and the supernatant was taken. The kit was removed from the 4 °C environment and balanced at room temperature for 25 min. The standard wells, sample wells, and blank wells were set, and the absorbance (OD) of each well was measured at 450 nm by zero-setting the blank well.

### 4.7. Statistics

Data were analyzed using the Origin Pro 9.0 software (Origin Lab, Northampton, MA, USA). Datasets were normalized to test the statistical significance of the treatment effect and a one-way ANOVA was performed using the SPSS 22.0 software (IBM, USA). Significant differences between group means were determined as *p* < 0.05 via Tukey’s and Fisher’s tests. 

## 5. Conclusions

In this study, we reconstructed the biosynthesis pathway of glycine betaine in the economically important seaweed *P. yezoensis* and experimentally detected the existence and rapid accumulation of GB as well as the transcriptional regulation under desiccation stress. The extensive illustration of the genetic foundation in the GB biosynthesis in *P. yezoensis* will undoubtedly further our understanding of the adaptive strategies of intertidal seaweeds in their evolutionary history and provide precious genetic resources for genetic engineering of algae and plants for abiotic stress tolerance. 

## Figures and Tables

**Table 1:** Choline dehydrogenase (CDH) genes in *P. yezoensis.*

Gene	GenBank Entry	ORF (bp)	Amino Acids	Conserved Domain
**PyCDH1**	MK294537	3092	679	GMC-oxred_N
**PyCDH2**	MK294538	1761	586	GMC-oxred_N
**PyCDH3**	MK294539	2289	762	GMC-oxred_N
**PyCDH4**	MK294540	1710	557	GMC-oxred_N
**PyCDH5**	MK294541	1955	569	GMC-oxred_N
**PyCDH6**	MK294542	2291	572	GMC-oxred_N
**PyCDH7**	MK294543	1620	539	GMC-oxred_N

**Table 2:** Information of primers.

Primer Name	GenBank Entry	Sequence Information (5′–3′)
BADH	MK294535	F: GCGTCCCTGCGAGCCACTCAC R: CCGTGTCAAAGGGGATAACCGT
PEAMT	MK294536	F: CTCTTCGCACCCGTGACCTG R: TGTCCAGGTAGGCGTCCGAG
CDH-1	MK294537	F: GAACCGTTTTCGCCCTATCGC R: CGCCACGCCCTTGACCC
CDH-2	MK294538	F: CCGCATTGTCTGGGCTGCA R: GACGCATCAACCACCCACAAGT
CDH-3	MK294539	F: GCGGTGGGCACCTGCCGGAT R: GGCGTTGGTGTTGCCACTCC
CDH-4	MK294540	F: CCGAGTGACCACAGGCGA R: AGCAGGTTGGTCTCCACACG
UBC	ACI47322.1	F: TCACAACGAGGATTTACCACC R: GAGGAGCACCTTGGAAACG
